# Key factors which concur to the correct therapeutic evaluation of herbal products in free radical-induced diseases

**DOI:** 10.3389/fphar.2015.00086

**Published:** 2015-04-22

**Authors:** Cesare Mancuso

**Affiliations:** Institute of Pharmacology, Catholic University School of MedicineRoma, Italy

**Keywords:** dietary antioxidants, free radical-induced diseases, herbal products, nutraceuticals, pharmacology

## Abstract

For many years now the world’s scientific literature has been perfused with articles on the therapeutic potential of natural products, the vast majority of which have herbal origins, as in the case of free radical-induced diseases. What is often overlooked is the effort of researchers who take into consideration the preclinical and clinical evaluation of these herbal products, in order to demonstrate the therapeutic efficacy and safety. The first critical issue to be addressed in the early stages of the preclinical studies is related to pharmacokinetics, which is sometimes not very favorable, of some of these products, which limits the bioavailability after oral intake. In this regard, it is worthy underlining how it is often unethical to propose the therapeutic efficacy of a compound on the basis of preclinical results obtained with far higher concentrations to those which, hopefully, could be achieved in organs and tissues of subjects taking these products by mouth. The most widely used approach to overcome the problem related to the low bioavailability involves the complexation of the active ingredients of herbal products with non-toxic carriers that facilitate the absorption and distribution. Even the induction or inhibition of drug metabolizing enzymes by herbal products, and the consequent variations of plasma concentrations of co-administered drugs, are phenomena to be carefully evaluated as they can give rise to side-effects. This risk is even greater when considering that people lack the perception of the risk arising from an over use of herbal products that, by their very nature, are considered risk-free.

## Introduction

For many years the use of natural products derived from herbs (thereafter “herbal products”), has been proposed as adjuvant therapy in diseases in which free radicals play a key role on the pathogenesis, e.g., neurodegenerative diseases, cancer, diabetes mellitus, and cardiovascular diseases (Qi et al., 2010; Mancuso et al., 2012a; Ergin et al., 2013). The reason for scientists’ interest in herbal products is linked to the fact that a number of preclinical studies have demonstrated the antioxidant and cytoprotective activity of these compounds (Brambilla et al., 2008; Calabrese et al., 2010; Mancuso et al., 2012a; Mancuso and Santangelo, 2014). Indeed, the data on some relevant clinical studies summarized in recent reviews and meta-analyzes have argued, partially or in some cases totally, the evidence obtained from studies of cellular systems or in laboratory animals on alleged therapeutic activity of herbal products. The aim of this article is not to provide an analytical review of the role played by herbal products as adjuvants in the treatment of the aforementioned pathologies (the interested reader may find numerous overviews that extensively examine the biological actions of any product), yet to analyze critically the main factors that should be taken into account when deciding on the therapeutic potential of a product derived from herbs, from the preclinical studies in animal models to clinical results. Each of these factors will be discussed considering, for example, some of the herbal products that yield sufficient preclinical and clinical pharmacology data.

As herbal products taken with food or contained in supplements are different from “prescription drugs” derived from plants (e.g., atropine, digitalis, morphine), they are often referred to as dietary supplements. In light of this, for the purpose of this paper, the terms “herbal products” and “dietary supplement” will be considered equivalent.

## Pharmacokinetic Factors

### Absorption and Distribution

**FIGURE 1:**
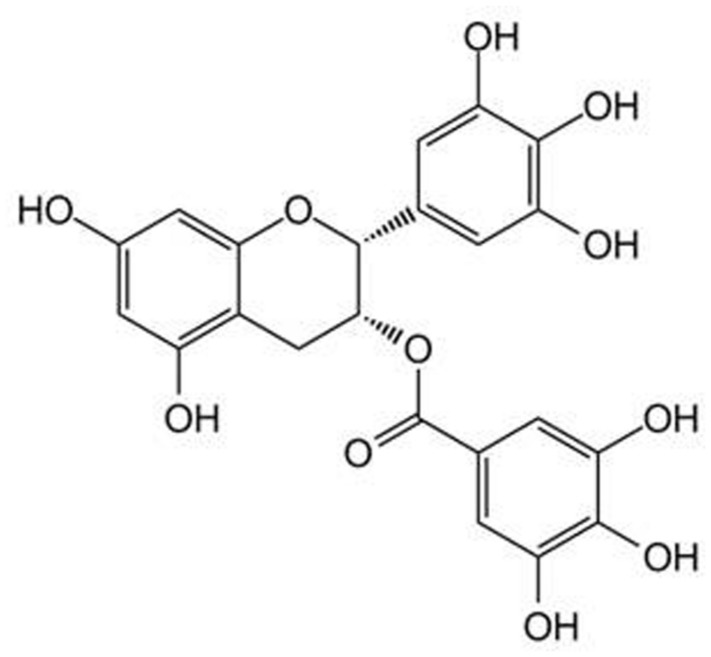
**Chemical structure of (–)-epigallocatechin gallate (EGCG)**.

**FIGURE 2:**
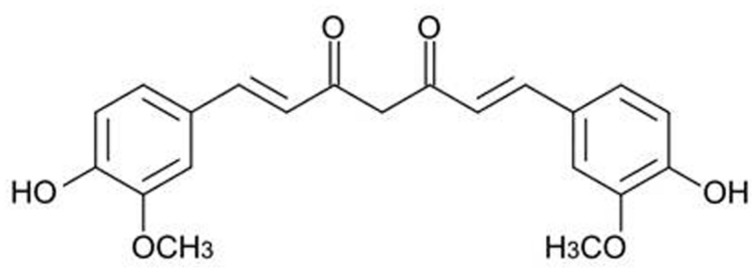
**Chemical structure of curcumin**.

**FIGURE 3:**
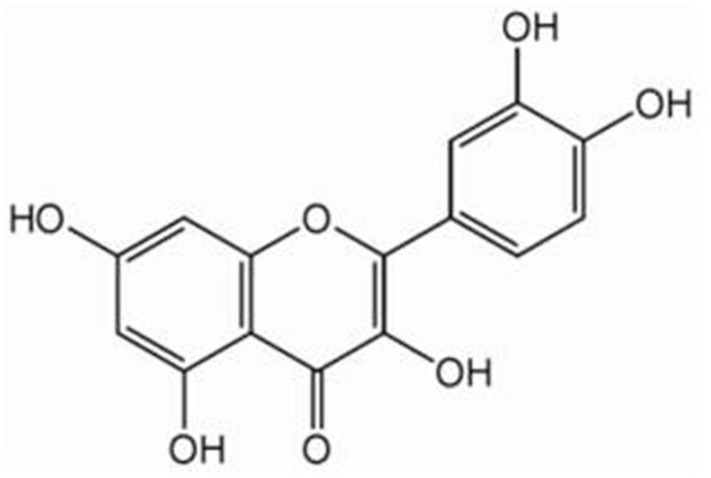
**Chemical structure of quercetin (QCT)**.

Because of their chemical structure, several herbal products possess a low solubility and stability in water solution or are quickly deteriorated in body fluids whose pH is neutral. (–)-Epigallocatechin gallate (EGCG, **Figure 1**), a polyphenol belonging to the subgroup of flavanols and particularly abundant in green tea, has a stability in water solution and at the body temperature of 37∘C concentration-dependent which gradually decreases with the decrease in the concentration of ECGC and with the contact of the pH of the solution to 7.4 (Proniuk et al., 2002). For these reasons, under physiological conditions, and to the concentrations that are likely to occur in the blood and tissues, the amounts of ECGC are neglectable. Similarly, the polyphenols curcumin (CUR, contained in the rhizome of *Curcuma longa Linn*., **Figure 2**) and quercetin (QCT, abundant in capers, berries, apples, onions, and other leafy green vegetables, **Figure 3**), in view of their high hydrophobicity, they have a water solubility of about to 1 μg/ml and 2 mg/ml, respectively (Srinivas et al., 2010; Zhang et al., 2011). Due to these physical–chemical characteristics, EGCG, CUR, and QCT are poorly absorbed after ingestion and have a very low bioavailability (Wang et al., 2014). This limitation in the absorption has complicated the ability of clinical trials to evaluate the efficacy of these natural compounds in numerous diseases, thereby preventing the accumulation of evidence about their utility or not for man. An effective strategy to increase the bioavailability of dietary supplements consists in their complexation with various types of nanoparticles or polysaccharides, e.g., chitosan and tri-methyl-chitosan (Wang et al., 2014). This technology has been recently applied to ECGC, CUR, and QCT which were produced in new formulations that have made a significant improvement in bioavailability; **Table 1** shows the variation of the main pharmacokinetic parameters of EGCG, CUR, and QCT complexed with different types of nanoparticles or polysaccharides. A careful analysis of **Table 1** shows the complexation of CUR and QCT with the different types of carriers increases markedly both the maximum peak concentration (*C*_max_) and the necessary time to reach it (*T*_max_), which suggests a slower, yet more effective absorption of the active ingredient. Simultaneously, the increase in the area under the curve (AUC) demonstrates how the presence of nanoparticles or particles derived from chitosan is capable of improving the bioavailability and this is particularly evident in the case of CUR and QCT. The increase in half-life (T_1/2_) in the case of formulations based on lipid carriers and polysaccharides implies an extension of the time of persistence of the active ingredient in the body and, therefore, a more prolonged pharmacological action. It is worthy emphasizing that the solid–lipid nanoparticles (SLN) have proved particularly effective carriers both for the CUR that QCT, thus increasing the bioavailability of many degrees of magnitude. As regards the EGCC, the formulation with the derivatives of the chitosan has proved able to increase the AUC by over 50% compared to an increase in *C*_max_ by only 10%.

**Table 1:** Summary about the complexation-induced modifications on the main pharmacokinetic parameters of some active ingredients of herbal products.

Compound/formulation	AUC	*C*_max_	*T*_max_	*T*_1/2_	Reference
ECGC	~116 nM⋅h^a^	~34.3 nM^a^	1.5 h^a^	3.4 h^a^	Dube et al. (2011)
Chitosan-NP-ECGC	~179 nM⋅h^a^	~37.8 nM^a^	1.5 h^a^		Dube et al. (2011)
Curcumin (CUR)	~312 ng/ml⋅h^b^	~245 nM^b^	0.5 h^b^	~1.0 h^b^	Shaikh et al. (2009)
Curcumin-PLGA	~3224 ng/ml⋅h^c^	~710 nM^c^	2.0 h^c^		Shaikh et al. (2009)
Curcumin-TMC	~12760 ng/ml⋅h^d^	~3.3 μM^d^	2.0 h^d^	~12 h^d^	Ramalingam and Ko (2014)
Curcumin-SLN	~42000 ng/ml⋅h^e^	~38 μM^e^	0.5 h^e^		Kakkar et al. (2010)
Quercetin (QCT)	~56 μg/ml⋅h^f^	~6 μg/ml^f^	5 h^f^	6 h^f^	Li et al. (2009)
Quercetin-SLN	~324 μg/ml⋅h^f^	~12 μg/ml^f^	8 h^f^	18 h^f^	Li et al. (2009)

^a^Male Swiss outbred mice treated with a single oral dose of EGCG or Chitosan-NP-ECGC at the dose of 0.76 mg/kg.

^b^Male Sprague–Dawley rats treated with 250 mg/kg curcumin *per os.*

^c^Male Sprague–Dawley rats treated with 100 mg/kg curcumin-PLGA *per os.*

^d^Balb/c mice treated with 50 mg/kg curcumin-TMC *per os*.

^e^Male Wistar rats treated with 50 mg/kg curcumin-SLN *per os.*

^f^Male Wistar rats treated orally with quercetin or quercetin-SLN suspensions at the dose of 50 mg/kg.

*AUC, are under the curve; *C*_max_, peak plasma concentration; NP, nanoparticles; PLGA, poly(lactic-co-glycolic acid); SLN, solid lipid nanoparticles; *T*_max_, time to reach the *C*_max_; *T*_1/2_, half-life; TMC, *N*-trimethyl Chitosan.*

One aspect still to be evaluated when using lipid carriers or polysaccharides is the ability that they are able to “guide” the compound towards the target organs where the accumulation must occur and this is all the more complex as the organs are protected by specific anatomical structures. An example may be the central nervous system as the brain and spinal cord are protected by the blood–brain barrier (BBB), which reduces the penetration of many substances. In this case, the use of certain formulations has proven particularly effective. As demonstrated by Ramalingam and Ko (2014), chitosan is a useful vector for ensuring the accumulation of CUR in the cytosol and in the brain membranes of rat tissue which increases the chances of a possible use of this formulation in neurodegenerative diseases. Moreover, the formulation based on SLN, administered orally, or intravenously, allows a certain accumulation of CUR in the mouse brain (Kakkar et al., 2010).

A further issue to be considered when deciding to evaluate a preparation of nanoparticles, particularly when administered for prolonged periods, is the possible toxicity of the carriers with which the active ingredients are complexed. The use of nanoparticles produced using indigestible oils (e.g., mineral oils) rather than digestible ones (e.g., corn oil) should be avoided because those produced using the indigestible oils may have a considerable growth in size due to the aggregation in the passage from the mouth to the stomach and to the intestine and this may cause a degree of toxicity for those who take it (McClements, 2013; Rao et al., 2013). Furthermore, it is always advisable to avoid the use of vectors in the preparation of which synthetic surfactants have been used (e.g., sorbitan esters and their ethoxylates) or organic solvents (e.g., acetone, hexane) although, currently, there are no reliable data about the quantity of these substances in the final product compared to those used initially for the preparation and, therefore, it is not possible to determine whether they are comprised within the range of toxicity. This consideration is particularly true for organic solvents which are also used, for example, in moderate amounts during the preparation of nanoparticles, but which are subsequently evaporated and only traces of which may remain in the finished product (McClements, 2013). Moreover, the diameter of the nanoparticles is a factor that could determine side effects. There is an inverse relationship between the diameter of the nanoparticles and extent of absorption as the smaller their diameter (usually between 100 and 1000 nm), the greater the possibility that they are absorbed by increasing the intracellular concentration of the active ingredient they contain. Thus, it is worthy considering that, in case of nanoparticles with a very small diameter, for example, very useful to facilitate the passage through the BBB, oral administration can result in a significant absorption at the oral, esophageal, or gastric level, reducing the amount of active ingredient that reaches the small intestine which is the site where absorption occurs. This could lead to the onset of the local, rather than systemic effects (McClements, 2013). In the case of nanoparticles administered by IV (e.g., liposomes), whose primary use is a carrier of antineoplastic drugs and does not seem relevant in the case of herbal products, it is worthy considering the risk of non-allergic hypersensitivity reactions due to complement activation (Wacker, 2013).

The influence of the processes of absorption and distribution in determining the bioavailability and blood and tissue concentrations of a dietary supplement, points out, if proof were needed, the importance of the concentrations achieved compared to the doses administered, a factor that not always guarantees the shift of the results from preclinical studies to man. In this regard, the case of the CUR is exemplary. Due to its low lipophilicity and low bioavailability (for an extensive review on the pharmacokinetics of CUR, see Calabrese et al., 2008), CUR must be administered in high doses to a maximum of 8 g/day. Clinical studies have shown that the administration of CUR at doses of 1–4 g/day PO for 6 months is able to determine plasma concentrations of 250–270 nM within 4 h from administration which are reduced to 60 nM after 24 h (Baum et al., 2008). When administered for a shorter time, as in the study by Garcea et al. (2004) in which CUR was administered at doses of 450–3600 mg/day PO for 1 week in subjects with colorectal cancer, the plasma concentration decreased to 3 nM. Regarding a supramaximal dose of CUR (10–12 g single dose orally to healthy volunteers), the plasma concentration achieved was around 160 nM (Lao et al., 2006). As regards tissue concentrations, the data available are rather limited. In patients with colorectal cancer and treated with CUR 1.8 to 3.6 g/day for 7 days, concentrations in colorectal tumor tissue and normal tissue were, respectively ~7 and 20 nmol/g (Garcea et al., 2005). Based on these concentrations, it is difficult to imagine the effects in humans of CUR that, in several preclinical models, has proven effective in the range 5–50 μM (Garcea et al., 2005; Calabrese et al., 2008; Mancuso et al., 2012a), i.e., concentrations approximately 25–250 times higher than those in plasma; the calculation compared to the concentrations of CUR in the tissues is more difficult, and may appear less accurate, although the order of magnitude may be the same or even greater.

### Metabolism and Elimination

The possibility that dietary supplements interact with drug metabolizing enzymes is no longer a matter of debate. Among these enzymes there are isoforms of cytochrome P450 (CYP) and UDP-glucuronosyltransferases (UGT) and sulfotransferases (SULT). The several isoforms of CYP account for phase I metabolism, whereas both UGT and SULT are responsible for phase II reactions. In most cases, both phase I and phase II enzymes increase the hydrophilicity of drugs promoting their elimination, while only a small number of drugs, so-called pro-drugs, needs activation, usually mediated by CYP, to give rise to a pharmacological action (Rautio et al., 2008). The extensive discussion of interactions among herbal products with drug metabolizing enzymes is out of the scope of this article, interested readers can refer to many comprehensive reviews in the literature on this topic (Na et al., 2011; Gurley et al., 2012; Hermann and von Richter, 2012).

Numerous studies have been conducted to evaluate the effects of dietary supplements on drug metabolizing enzymes, although not always the results were consistent. There are several reasons that do not always ensure univocity of evidence, including the different experimental systems used (e.g., the use of cell lines vs. laboratory animals vs. clinical studies), the doses administered and the resulting blood/tissue concentrations reached, above all, the use of individual components of the multicomponent herbal products versus extracts. A typical example is the EGCG that in studies of human hepatocytes or immortalized cell lines induced the CYP1A1 isoform, while in S. *typhimurium* mutagenesis assay it noncompetitively inhibited CYP1A1, CYP2C9, and CYP3A4 (Williams et al., 2000; Allen et al., 2001; Muto et al., 2001; Shord et al., 2009). Green tea extracts also inhibited the activity of SULT 1A1 and SULT1A3 in a recombinant model of human SULT (Nishimuta et al., 2007; Nagai et al., 2009). Studies on rodents supplemented with various types of green tea showed a moderate induction of CYP1A and UGT in the liver (Maliakal and Wanwimolruk, 2001; Niwattisaiwong et al., 2004; Jang et al., 2005). Lastly, 42 healthy volunteers supplemented with 200 mg EGCG orally for 4 weeks showed a small yet significant reduction in CYP3A4 activity (Chow et al., 2006). These results were not confirmed in another study in which 11 healthy volunteers were treated for 14 days with two capsules/day of green tea containing 200 mg of EGCG, but in the absence of caffeine. This last datum further confirms the confounding role of any additional components in of the raw extracts of herbal products and not directly related to the purified active principle(s) (Donovan et al., 2004). The possible confounding role of additional components in the preparations obtained from the herbal raw material could also affect the results of clinical trials designed to evaluate the effectiveness of herbal products. The intake of purified active principles allow, for instance, an accurate titration of the administered dose versus the therapeutic effects and would be of great help in the identification of specific clinical outcomes, thus making the design of clinical trials more effective.

## Pharmacodynamic Factors

**FIGURE 4:**
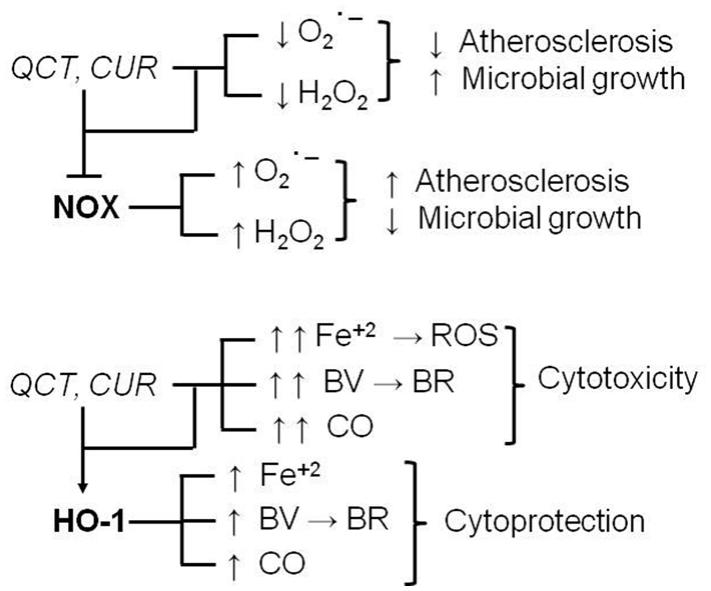
**Chronic supplementation with quercetin (QCT) and curcumin (CUR): dual pharmacodynamic effects due to the interaction with NADPH oxidase (NOX) and heme oxygenase-1 (HO-1).** For further information see text. BR, bilirubin; BV, biliverdin; CO, carbon monoxide; ROS, reactive oxygen species; Fe^+2^, ferrous iron; H_2_O_2_, hydrogen peroxide; $O_{2}^{∙\_}$; superoxide anion, –I, inhibition.

In recent years, numerous studies have shown dietary supplements are able to regulate many intracellular pathways involved in the mechanisms of cytoprotection/cytotoxicity. Several papers are available in literature showing inhibitory interactions between herbal products and clearly cytotoxic signaling systems [e.g., the inducible isoforms of nitric oxide synthase (iNOS) or cyclooxygenase (COX-2), NADPH oxidase] and stimulatory interactions on cytoprotective signaling systems [e.g., superoxide dismutases, catalase, heme oxygenase-1 (HO-1), etc.]. However, one aspect is not always rightly pointed out and on which more and more attention should be focused in the future, is the assessment of some of these cellular systems with borderline effects, that are cytoprotective or cytotoxic on the basis of the type of cell or tissue and their redox state. For example, NADPH oxidase is a membrane enzyme that, in the presence of NADPH, is able to transfer electrons to molecular oxygen generating free radicals, such as superoxide anion, which, in some compartments, is further converted to hydrogen peroxide (for an extensive review see Seifert and Schultz, 1991). Due to its ability to generate ROS, NADPH oxidase is considered to play a key role in atherosclerosis, and inhibitors of these enzyme have been shown to counteract plaque formation (Meyer and Schmitt, 2000; Yokoyama et al., 2000). On the other hand, in phagosomes NADPH oxidase-derived superoxide and secondary ROS play an anti-infective role because they contribute to kill bacteria and fungi (Segal, 2005). Therefore, dietary supplements, such as QCT and CUR, which reduce NADPH oxidase activity in several preclinical models of atherosclerosis are considered potential adjuvant agents in the prevention or chronic treatment of cardiovascular diseases (Deby-Dupont et al., 2005; Sánchez et al., 2006; Romero et al., 2009). On the other hand, the NADPH oxidase inhibition mediated by these natural compounds could decrease ROS formation in fagosomes, thus reducing microbial killing and transforming these compounds in pro-infective agents (**Figure 4**). Similarly, the induction of HO-1 is considered an early protective event in the cell stress response because it degrades heme into carbon monoxide, ferrous iron and biliverdin which is further transformed, through the biliverdin reductase activity, into the powerful free radical scavenger bilirubin (Maines, 1997; Barone et al., 2009; Mancuso and Barone, 2009a; Mancuso et al., 2012b). However, a sustained induction of HO-1, which could occur in the event of prolonged supplementation with CUR, QCT, and other herbal products, could be harmful because it (i) depletes cells of heme, which is necessary as co-factor of several proteins, (ii) further increases the release of both bilirubin and carbon monoxide, which become *per se* toxic if produced in excess, and ferrous iron which triggers Fenton reaction (Winterbourn, 1995) and generates ROS, this latter being responsible for both lipid and protein oxidation and cell death (**Figure 4**; Tang et al., 2013; Murphy et al., 2014).

## Safety Issues

On the basis of the above views, the induction or inhibition of drug metabolizing enzymes by herbal products and the relative changes of hemoconcentrations of any co-administered drugs, are important factors that contribute to the onset of side effects. As previously explained, the literature data related to changes of the enzyme activity of CYP, UGT, or SULT isoforms, are not always univocal, indeed, they are sometimes also contradictory and this has so far not allowed to state any definite theories about the necessity or convenience, to avoid associations between herbal products and certain medications. The main problems in this regard have arisen since the majority of the evidence to support derived from preclinical studies carried out on cell lines or laboratory animals without evidence of a direct effect on humans. For example, Golden et al. (2009) have shown that ECGC reduces the cytotoxic activity of bortezomib, a boronic acid derivative used to treat multiple myeloma or other malignancies, in multiple myeloma and glioblastoma cell lines. According to these results, some articles in the literature recommend avoiding the use of green tea in subjects treated with bortezomib (Mancuso and Barone, 2009b; Shah et al., 2009; Jia and Liu, 2013). Studies on rodents have shown that EGCG-related inhibition of CYP3A4 may increase blood concentrations of verapamil, diltiazem, or midazolam determining potential toxic effects (Muto et al., 2001; Li and Choi, 2008; Chung et al., 2009). In the same experimental model, the moderate induction of CYP1A by green tea determined a reduced effect for the antipsychotic drug clozapine (Jang et al., 2005). However, direct evidence was obtained from humans for a number of herbal products, thus providing unequivocal demonstrations of toxic interactions with drugs. Ginkgo biloba is a herbal product with an excellent bioavailability after oral administration (Kleijnen and Knipschild, 1992), initially considered useful as adjuvant treatment in dementia and consequently dropped due to lack of clinical confirmations (for a recent review see Mancuso et al., 2012a). Chronic administration of ginkgo biloba, which induces CYP2C19, significantly reduced the blood amounts, and therefore the clinical efficacy, of omeprazole particularly in individuals considered poor metabolizers (Yin et al., 2004). Moreover, because of the activation of CYP2C19, ginkgo biloba has reduced the blood concentrations of valproic acid and phenytoin in an epileptic subject causing fatal seizures (Kupiec and Raj, 2005). Lastly, ginkgo biloba has caused the state of coma in an 80-year-old individual suffering from Alzheimer’s disease and treated with trazodone (Galluzzi et al., 2000). This last adverse effect was shown to be related to the ginkgo biloba-mediated stimulation of CYP3A4 activity with marked transformation of trazodone into the active metabolite 1- (*m*-chlorophenyl) piperazine which increased the GABAergic tone in the brain and precipitated coma (Galluzzi et al., 2000).

Another exception must be made for the grapefruit juice for which there is clinical evidence of dangerous drug interactions. Indeed, grapefruit is rich in furanocoumarins, also well absorbed following grapefruit intake (Uesawa et al., 2008; Messer et al., 2011) which are responsible for the inhibition of CYP3A4 isoform responsible for the metabolism of many drugs. The intake through a diet grapefruit has increased the blood concentrations of ethinylestradiol and caused deep venous thrombosis from the external iliac vein distal to the calf veins (Grande et al., 2009) in a 42-year-old woman. The intake of grapefruit juice is not recommended in patients treated with simvastatin and lovastatin because of a high risk of toxic effects, such as rhabdomyolysis (Dreier and Endres, 2004; Neuvonen et al., 2008). The dietary intake of grapefruit juice increased the hemoconcentrations of nifedipine and caused significant hypotensive effects in a 50-year-old man with essential hypertension (Nakagawa and Goto, 2010).

## Conclusion

The above considerations highlight the difficulties faced by researchers when deciding on the possibility or not to start studying the herbal-derived products as adjuvants to conventional therapies for many diseases in which free radicals play a prevalent pathogenic role. Some problems, such as those related to the absorption and distribution of herbal products can be overcome with the help of medicinal chemistry that allows to enhance better absorbed formulations when administered orally, and to reach high concentrations of active ingredient in target organs and tissues. Unfortunately, to date there are no sufficient clinical studies demonstrating the efficacy and safety of these new formulations; in this respect, greater interaction is advocated among researchers who are committed to the preparation and preclinical evaluation of these formulations, often belonging to non-profit institutions, with the pharmaceutical industry, which would have the financial resources to design and implement clinical trials.

The problems stemming from the modulation by herbal products of drug metabolizing enzymes are difficult to manage. What makes it even more difficult is to avoid side-effects arising from the simultaneous intake of medications, dietary or as a supplement, with herbal products and the almost total lack of risk perception by patients. Who would ever think that eating a grapefruit for breakfast or drinking 2–3 glasses of grapefruit juice a day (in particular in warm periods of the year) can pose a serious health risk in case of simultaneous intake of progestogens, statins, or nifedipine? An even more unacceptable risk when considering that the most affected could be vulnerable populations, such as, possibly, the elderly affected by heart disease. A serious approach to solving this problem would provide accurate information on potential risks arising from a prolonged use of herbal products, always recommending medical supervision and shelving the idea that everything is “natural” is “healthy” and “devoid of risks.”

## Conflict of Interest Statement

The Associate Editor Silvana Gaetani declares that, despite having collaborated with the author Cesare Mancuso, the review process was handled objectively and no conflict of interest exists. The author declares that the research was conducted in the absence of any commercial or financial relationships that could be construed as a potential conflict of interest.

## References

[B1] Allen S. W. Mueller L. Williams S. N. Quattrochi L. C. Raucy J. (2001). The use of a high-volume screening procedure to assess the effects of dietary flavonoids on human cyp1a1 expression. *Drug Metab. Dispos.* 29 1074–1079. 11454723

[B2] Barone E. Trombino S. Cassano R. Sgambato A. De Paola B. Di Stasio E. (2009). Characterization of the S-denitrosylating activity of bilirubin. *J. Cell. Mol. Med*. 13 2365–2375 10.1111/j.1582-4934.2009.00680.x 20141617 PMC9181359

[B3] Baum L. Lam C. W. Cheung S. K. Kwok T. Lui V. Tsoh J. (2008). Six-month randomized, placebo-controlled, double-blind, pilot clinical trial of curcumin in patients with Alzheimer disease. *J. Clin. Psychopharmacol.* 28 110–113 10.1097/jcp.0b013e318160862c 18204357

[B4] Brambilla D. Mancuso C. Scuderi M. R. Bosco P. Cantarella G. Lempereur L. (2008). The role of antioxidant supplement in immune system, neoplastic, and neurodegenerative disorders: a point of view for an assessment of the risk/benefit profile. *Nutr. J.* 7 29 10.1186/1475-2891-7-29 PMC2572622 18826565

[B5] Calabrese V. Bates T. E. Mancuso C. Cornelius C. Ventimiglia B. Cambria M. T. (2008). Curcumin and the cellular stress response in free radical-related diseases. *Mol. Nutr. Food Res.* 52 1062–1073 10.1002/mnfr.200700316 18792015

[B6] Calabrese V. Cornelius C. Trovato A. Cavallaro M. Mancuso C. Di Rienzo L. (2010). The hormetic role of dietary antioxidants in free radical-related diseases. *Curr. Pharm. Des.* 16 877–883 10.2174/138161210790883615 20388101

[B7] Chow H. H. Hakim I. A. Vining D. R. Crowell J. A. Cordova C. A. Chew W. M. (2006). Effects of repeated green tea catechin administration on human cytochrome P450 activity. *Cancer Epidemiol. Biomarkers Prev.* 15 2473–2476 10.1158/1055-9965.EPI-06-0365 17164372

[B8] Chung J. H. Choi D. H. Choi J. S. (2009). Effects of oral epigallocatechin gallate on the oral pharmacokinetics of verapamil in rats. *Biopharm. Drug Dispos.* 30 90–93 10.1002/bdd.644 19226653

[B9] Deby-Dupont G. Mouithys-Mickalad A. Serteyn D. Lamy M. Deby C. (2005). Resveratrol and curcumin reduce the respiratory burst of Chlamydia-primed THP-1 cells. *Biochem. Biophys. Res. Commun.* 333 21–27 10.1016/j.bbrc.2005.05.073 15939398

[B10] Donovan J. L. Chavin K. D. Devane C. L. Taylor R. M. Wang J. S. Ruan Y. (2004). Green tea (*Camellia sinensis*) extract does not alter cytochrome p450 3A4 or 2D6 activity in healthy volunteers. *Drug Metab. Dispos.* 32 906–908 10.1124/dmd.104.000083 15319329

[B11] Dreier J. P. Endres M. (2004). Statin-associated rhabdomyolysis triggered by grapefruit consumption. *Neurology* 62 670 10.1212/WNL.62.4.670 14981197

[B12] Dube A. Nicolazzo J. A. Larson I. (2011). Chitosan nanoparticles enhance the plasma exposure of (-)-epigallocatechin gallate in mice through an enhancement in intestinal stability. *Eur. J. Pharm. Sci.* 44 422–426 10.1016/j.ejps.2011.09.004 21925598

[B13] Ergin V. Bali E. B. Hariry R. E. Karasu C. (2013). Natural products and the aging process. *Horm. Mol. Biol. Clin. Investig.* 16 55–64 10.1515/hmbci-2013-0031 25436747

[B14] Galluzzi S. Zanetti O. Binetti G. Trabucchi M. Frisoni G. B. (2000). Coma in a patient with Alzheimer’s disease taking low dose trazodone and gingko biloba. *J. Neurol. Neurosurg. Psychiatry* 68 679–680 10.1136/jnnp.68.5.679a 10836866 PMC1736918

[B15] Garcea G. Berry D. P. Jones D. J. Singh R. Dennison A. R. Farmer P. B. (2005). Consumption of the putative chemopreventive agent curcumin by cancer patients: assessment of curcumin levels in the colorectum and their pharmacodynamic consequences. *Cancer Epidemiol. Biomarkers Prev.* 14 120–125. 15668484

[B16] Garcea G. Jones D. J. Singh R. Dennison A. R. Farmer P. B. Sharma R. A. (2004). Detection of curcumin and its metabolites in hepatic tissue and portal blood of patients following oral administration. *Br. J. Cancer* 90 1011–1015 10.1038/sj.bjc.6601623 14997198 PMC2409622

[B17] Golden E. B. Lam P. Y. Kardosh A. Gaffney K. J. Cadenas E. Louie S. G. (2009). Green tea polyphenols block the anticancer effects of bortezomib and other boronic acid-based proteasome inhibitors. *Blood* 113 5927–5937 10.1182/blood-2008-07-171389 19190249

[B18] Grande L. A. Mendez R. D. Krug R. T. Verschuyl E. J. (2009). Attention–grapefruit!. *Lancet* 373 1222 10.1016/S0140-6736(09)60289-0 19345832

[B19] Gurley B. J. Fifer E. K. Gardner Z. (2012). Pharmacokinetic herb-drug interactions (part 2): drug interactions involving popular botanical dietary supplements and their clinical relevance. *Planta Med.* 78 1490–1514 10.1055/s-0031-1298331 22565299

[B20] Hermann R. von Richter O. (2012). Clinical evidence of herbal drugs as perpetrators of pharmacokinetic drug interactions. *Planta Med*. 78 1458–1477 10.1055/s-0032-1315117 22855269

[B21] Jang E. H. Choi J. Y. Park C. S. Lee S. K. Kim C. E. Park H. J. (2005). Effects of green tea extract administration on the pharmacokinetics of clozapine in rats. *J. Pharm. Pharmacol.* 57 311–316 10.1211/0022357055687 15807986

[B22] Jia L. Liu F. T. (2013). Why bortezomib cannot go with ‘green’? *Cancer Biol. Med*. 10 206–213 10.7497/j.issn.2095-3941.2013.04.004 24349830 PMC3860349

[B23] Kakkar V. Singh S. Singla D. Sahwney S. Chauhan A. S. Singh G. (2010). Pharmacokinetic applicability of a validated liquid chromatography tandem mass spectroscopy method for orally administered curcumin loaded solid lipid nanoparticles to rats. *J. Chromatogr. B Analyt. Technol. Biomed. Life Sci.* 878 3427–3431 10.1016/j.jchromb.2010.10.017 21111692

[B24] Kleijnen J. Knipschild P. (1992). Ginkgo biloba. *Lancet* 340 1136–1139 10.1016/0140-6736(92)93158-J 1359218

[B25] Kupiec T. Raj V. (2005). Fatal seizures due to potential herb-drug interactions with Ginkgo biloba. *J. Anal. Toxicol.* 29 755–758 10.1093/jat/29.7.755 16419414

[B26] Lao C. D. Ruffin M. T. IV Normolle D. Heath D. D. Murray S. I. Bailey J. M. (2006). Dose escalation of a curcuminoid formulation. *BMC Complement*. *Altern. Med*. 6 10 10.1186/1472-6882-6-10 PMC1434783 16545122

[B27] Li C. Choi J. S. (2008). Effects of epigallocatechin gallate on the bioavailability and pharmacokinetics of diltiazem in rats. *Pharmazie* 63 815–818. 19069242

[B28] Li H. Zhao X. Ma Y. Zhai G. Li L. Lou H. (2009). Enhancement of gastrointestinal absorption of quercetin by solid lipid nanoparticles. *J. Control Release* 133 238–244 10.1016/j.jconrel.2008.10.002 18951932

[B29] Maines M. D. (1997). The heme oxygenase system: a regulator of second messenger gases. *Annu. Rev. Pharmacol. Toxicol.* 37 517–554 10.1146/annurev.pharmtox.37.1.517 9131263

[B30] Maliakal P. P. Wanwimolruk S. (2001). Effect of herbal teas on hepatic drug metabolizing enzymes in rats. *J. Pharm. Pharmacol.* 53 1323–1329 10.1211/0022357011777819 11697539

[B31] Mancuso C. Barone E. (2009a). The heme oxygenase/biliverdin reductase pathway in drug research and development. *Curr. Drug Metab.* 10 579–594 10.2174/138920009789375405 19702533

[B32] Mancuso C. Barone E. (2009b). Therapeutic use of tea derivatives: all that glitters is not gold. *Blood* 114 2359–2360 10.1182/blood-2009-07-231761 19745081

[B33] Mancuso C. Siciliano R. Barone E. Preziosi P. (2012a). Natural substances and Alzheimer’s disease: from preclinical studies to evidence based medicine. *Biochim. Biophys. Acta* 1822 616–624 10.1016/j.bbadis.2011.09.004 21939756

[B34] Mancuso C. Barone E. Guido P. Miceli F. Di Domenico F. Perluigi M. (2012b). Inhibition of lipid peroxidation and protein oxidation by endogenous and exogenous antioxidants in rat brain microsomes in vitro. *Neurosci. Lett.* 518 101–105 10.1016/j.neulet.2012.04.062 22609281

[B35] Mancuso C. Santangelo R. (2014). Ferulic acid: pharmacological and toxicological aspects. *Food Chem. Toxicol.* 65 185–195 10.1016/j.fct.2013.12.024 24373826

[B36] McClements D. J. (2013). Edible lipid nanoparticles: digestion, absorption, and potential toxicity. *Prog. Lipid Res.* 52 409–423 10.1016/j.plipres.2013.04.008 23664907

[B37] Messer A. Nieborowski A. Strasser C. Lohr C. Schrenk D. (2011). Major furocoumarins in grapefruit juice I: levels and urinary metabolite(s). *Food Chem. Toxicol.* 49 3224–3231 10.1016/j.fct.2011.09.005 21945416

[B38] Meyer J. W. Schmitt M. E. (2000). A central role for the endothelial NADPH oxidase in atherosclerosis. *FEBS Lett.* 472 1–4 10.1016/S0014-5793(00)01397-1 10781793

[B39] Murphy A. Testa K. Berkelhammer J. Hopkins S. Loo G. (2014). Impact of antioxidants on the ability of phenolic phytochemicals to kill HCT116 colon cancer cells. *Free Radic. Res.* 48 313–321 10.3109/10715762.2013.867958 24256565

[B40] Muto S. Fujita K. Yamazaki Y. Kamataki T. (2001). Inhibition by green tea catechins of metabolic activation of procarcinogens by human cytochrome P450. *Mutat. Res.* 479 197–206 10.1016/S0027-5107(01)00204-4 11470492

[B41] Na D. H. Ji H. Y. Park E. J. Kim M. S. Liu K. H. Lee H. S. (2011). Evaluation of metabolism-mediated herb-drug interactions. *Arch. Pharm. Res.* 34 1829–1842 10.1007/s12272-011-1105-0 22139684

[B42] Nagai M. Fukamachi T. Tsujimoto M. Ogura K. Hiratsuka A. Ohtani H. (2009). Inhibitory effects of herbal extracts on the activity of human sulfotransferase isoform sulfotransferase 1A3 (SULT1A3). *Biol. Pharm. Bull.* 32 105–109 10.1248/bpb.32.105 19122289

[B43] Nakagawa K. Goto T. (2010). Effects of ingestion of grapefruit juice or grapefruit on the hypotensive effect and plasma concentrations of dihydropyridine calcium antagonists (amlodipine and nifedipine): a case study. *Clin. Exp. Hypertens.* 32 71–75 10.3109/10641960902960540 20374180

[B44] Neuvonen P. J. Backman J. T. Niemi M. (2008). Pharmacokinetic comparison of the potential over-the-counter statins simvastatin, lovastatin, fluvastatin and pravastatin. *Clin. Pharmacokinet.* 47 463–474 10.2165/00003088-200847070-00003 18563955

[B45] Nishimuta H. Ohtani H. Tsujimoto M. Ogura K. Hiratsuka A. Sawada Y. (2007). Inhibitory effects of various beverages on human recombinant sulfotransferase isoforms SULT1A1 and SULT1A3. *Biopharm. Drug Dispos.* 28 491–500 10.1002/bdd.579 17876860

[B46] Niwattisaiwong N. Luo X. X. Coville P. F. Wanwimolruk S. (2004). Effects of Chinese, Japanese and Western tea on hepatic P450 enzyme activities in rats. *Drug Metabol. Drug Interact.* 20 43–56 10.1515/DMDI.2004.20.1-2.43 15283302

[B47] Proniuk S. Liederer B. M. Blanchard J. (2002). Preformulation study of epigallocatechin gallate, a promising antioxidant for topical skin cancer prevention. *J. Pharm. Sci.* 91 111–116 10.1002/jps.10009 11782902

[B48] Qi L. W. Liu E. H. Chu C. Peng Y. B. Cai H. X. Li P. (2010). Anti-diabetic agents from natural products–an update from 2004 to 2009. *Curr. Top. Med. Chem*. 10 434–457 10.2174/156802610790980620 20180758

[B49] Ramalingam P. Ko Y. T. (2014). Enhanced oral delivery of curcumin from N-trimethyl chitosan surface-modified solid lipid nanoparticles: pharmacokinetic and brain distribution evaluations. *Pharm. Res.* 32 389–402 10.1007/s11095-014-1469-1 25082210

[B50] Rao J. Decker E. A. Xiao H. McClements D. J. (2013). Nutraceutical nanoemulsions: influence of carrier oil composition (digestible versus indigestible oil) on β-carotene bioavailability. *J. Sci. Food Agric.* 93 3175–3183 10.1002/jsfa.6215 23649644

[B51] Rautio J. Kumpulainen H. Heimbach T. Oliyai R. Oh D. Järvinen T. (2008). Prodrugs: design and clinical applications. *Nat. Rev. Drug Discov.* 7 255–270 10.1038/nrd2468 18219308

[B52] Romero M. Jiménez R. Sánchez M. López-Sepúlveda R. Zarzuelo M. J. O’Valle F. (2009). Quercetin inhibits vascular superoxide production induced by endothelin-1: role of NADPH oxidase, uncoupled eNOS and PKC. *Atherosclerosis* 202 58–67 10.1016/j.atherosclerosis.2008.03.007 18436224

[B53] Sánchez M. Galisteo M. Vera R. Villar I. C. Zarzuelo A. Tamargo J. (2006). Quercetin downregulates NADPH oxidase, increases eNOS activity and prevents endothelial dysfunction in spontaneously hypertensive rats. *J. Hypertens.* 24 75–84 10.1097/01.hjh.0000198029.22472.d9 16331104

[B54] Segal A. W. (2005). How neutrophils kill microbes. *Annu. Rev. Immunol.* 23 197–223 10.1146/annurev.immunol.23.021704.115653 15771570 PMC2092448

[B55] Seifert R. Schultz G. (1991). The superoxide-forming NADPH oxidase of phagocytes. An enzyme system regulated by multiple mechanisms. *Rev. Physiol. Biochem. Pharmacol.* 117 1–338. 1659735

[B56] Shah J. J. Kuhn D. J. Orlowski R. Z. (2009). Bortezomib and EGCG: no green tea for you? *Blood* 113 5695–5696 10.1182/blood-2009-03-204776 19498025 PMC2700310

[B57] Shaikh J. Ankola D. D. Beniwal V. Singh D. Kumar M. N. (2009). Nanoparticle encapsulation improves oral bioavailability of curcumin by at least 9-fold when compared to curcumin administered with piperine as absorption enhancer. *Eur. J. Pharm. Sci.* 37 223–230 10.1016/j.ejps.2009.02.019 19491009

[B58] Shord S. S. Shah K. Lukose A. (2009). Drug-botanical interactions: a review of the laboratory, animal, and human data for 8 common botanicals. *Integr. Cancer Ther.* 8 208–227 10.1177/1534735409340900 19815591

[B59] Srinivas K. King J. W. Howard L. R. Monrad J. K. (2010). Solubility and solution thermodynamic properties of quercetin and quercetin dihydrate in subcritical water. *J. Food Eng.* 100 208–218 10.1016/j.jfoodeng.2010.04.001

[B60] Tang Y. Tian H. Shi Y. Gao C. Xing M. Yang W. (2013). Quercetin suppressed CYP2E1-dependent ethanol hepatotoxicity via depleting heme pool and releasing CO. *Phytomedicine* 20 699–704 10.1016/j.phymed.2013.03.010 23583009

[B61] Uesawa Y. Yamada H. Mohri K. (2008). Determination of bergamottin in human plasma after grapefruit juice ingestion by an UPLC/MS/MS method. *Pharmazie* 63 110–112 10.1691/ph.2008.7289 18380396

[B62] Wacker M. (2013). Nanocarriers for intravenous injection–the long hard road to the market. *Int. J. Pharm.* 457 50–62 10.1016/j.ijpharm.2013.08.079 24036012

[B63] Wang S. Su R. Nie S. Sun M. Zhang J. Wu D. (2014). Application of nanotechnology in improving bioavailability and bioactivity of diet-derived phytochemicals. *J. Nutr. Biochem.* 25 363–376 10.1016/j.jnutbio.2013.10.002 24406273 PMC3959237

[B64] Williams S. N. Shih H. Guenette D. K. Brackney W. Denison M. S. Pickwell G. V. (2000). Comparative studies on the effects of green tea extracts and individual tea catechins on human CYP1A gene expression. *Chem. Biol. Interact.* 128 211–229 10.1016/S0009-2797(00)00204-0 11064004

[B65] Winterbourn C. C. (1995). Toxicity of iron and hydrogen peroxide: the Fenton reaction. *Toxicol. Lett.* 82–83, 969–974 10.1016/0378-4274(95)03532-X 8597169

[B66] Yin O. Q. Tomlinson B. Waye M. M. Chow A. H. Chow M. S. (2004). Pharmacogenetics and herb-drug interactions: experience with Ginkgo biloba and omeprazole. *Pharmacogenetics* 14 841–850 10.1097/00008571-200412000-00007 15608563

[B67] Yokoyama M. Inoue N. Kawashima S. (2000). Role of the vascular NADH/NADPH oxidase system in atherosclerosis. *Ann. N. Y. Acad. Sci.* 902 241–247; discussion 247–248 10.1111/j.1749-6632.2000.tb06319.x 10865844

[B68] Zhang F. Koh G. Y. Jeansonne D. P. Hollingsworth J. Russo P. S. Vicente G. (2011). A novel solubility-enhanced curcumin formulation showing stability and maintenance of anticancer activity. *J. Pharm. Sci.* 100 2778–2789 10.1002/jps.22512 21312196

